# An unusual evolution of a partitioned post tubercular meningitic hydrocephalus

**DOI:** 10.11604/pamj.2020.36.209.21342

**Published:** 2020-07-23

**Authors:** Hilal Abboud, Abdessamad Elouahabi

**Affiliations:** 1 Department of Neurosurgery, Specialty Hospital-ONO, Mohamed V University, Rabat, Morocco

**Keywords:** Calcification, unusual evolution, partitioned hydrocephalus, tuberculous meningitis

## Image in medicine

Post meningitic hydrocephalus occurs in 20 to 70% of bacterial or BK meningitis in infants and are responsible for a severe neuropsychic sequelae, the compartmentalized forms are seen in less than 2% of cases, and can pose therapeutic difficulties for drainage of large or multiple partitions. Calcification, rather a mode of involution of the fleshy lesions, is an unusual evolutionary mode of partitioned hydrocephalus. We report the case of a 26 years old man, treated at the age of six months for compartmentalized hydrocephalus on tuberculous meningitis with a good outcome under VPS. Recent cerebral magnetic resonance imaging (MRI) and computed tomography (CT) scan (A,B,C and D) after an epileptic episode found a sequellar ventriculomegaly in addition to an intracerebral, right occipital, cystic formation with complete calcification of the wall, revealing an exceptional evolutionary mode of compartmentalized hydrocephalus. Given the good control of epilepsy under medical treatment, the surgical indication was not retained.

**Figure 1:**
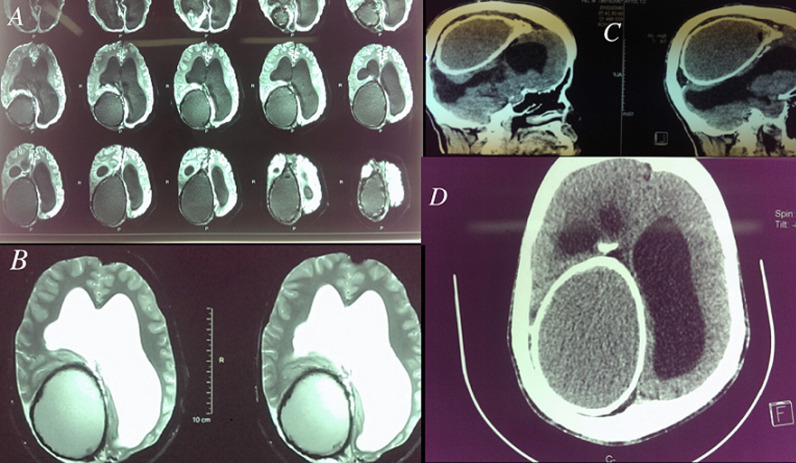
A) FLAIR- weighted cerebral MRI on axial sequences: a sequellar ventriculomegaly without signs of transependymal resorption, in addition to an intracerebral, right occipital, oval formation with a complete calcification of the wall; B) T2- weighted cerebral MRI on axial sequences: the oval formation seems to be extra axial with a fluid density, but different from the CSF signal; C) cerebral CT scan on parenchyma window and sagittal sequences: despite the large volume of the oval formation, there is no perilesional edema, in favour of the age of evolution; D) cerebral CT scan on parenchyma window and axial sequences: a bone bulge adjacent to the calcified cyst; the proximal tip of the catheter of VPS Shunt is seen near the cyst

